# Cellulose mini-membranes modified with TiO_2_ for separation, determination, and speciation of arsenates and selenites

**DOI:** 10.1007/s00604-020-04387-4

**Published:** 2020-07-06

**Authors:** Beata Zawisza, Rafal Sitko, Ignasi Queralt, Eva Margui, Anna Gagor

**Affiliations:** 1 Institute of Chemistry, University of Silesia, Szkolna 9, 40-006 Katowice, Poland; 2 Department of Geosciences, Institute of Environmental Assessment and Water Research, IDAEA-CSIC, Jordi Girona St., 18-26, 08034 Barcelona, Spain; 3 Department of Chemistry, Faculty of Sciences, University of Girona, C/M.Aurèlia Campmany 69, Girona, Spain; 4 Institute of Low Temperature and Structure Research, Polish Academy of Sciences, P.O. Box 1410, 50-950 Wrocław, Poland

**Keywords:** Arsenate, Selenite, Preconcentration, Complexes, Titanium dioxide

## Abstract

Sorptive and selective mini-membranes based on TiO_2_ directly synthesized onto cellulose filters (TiO_2_@cellulose) have been developed. The in situ synthesis of TiO_2_@cellulose applied is simple and economically advantageous. The obtained membranes can be useful for (1) separating arsenic(V) and selenium(IV) from other ions and organic matter, (2) speciation of arsenic and selenium, and (3) determining ulratraces of these ions in water samples. The membranes exhibit good stability and high maximum adsorption capacities for Se(IV) (71 mg g^−1^) and As(V) (41 mg g^−1^). A monolayer chemical adsorption of analytes on the membranes was confirmed. The structure of membranes was examined with scanning electron microscopy, x-ray diffractometry, and micro energy-dispersive x-ray fluorescence spectrometry (μ-EDXRF). The membranes were characterized by homogenous distribution of TiO_2_ onto cellulose. The TiO_2_@cellulose was used as a new sorbent in micro-solid phase extraction for determination of Se(IV) and As(V) by EDXRF. Using direct analysis of mini-membranes after sorption of analytes avoids the elution step. Thus, the proposed procedure is an attractive and solvent-free option for quantitative monitoring of Se(IV) and As(V) in different materials. Both analytes were quantitatively and simultaneously separated/determined from samples at pH 2 with very good recovery (close to 100%), precision (4.5%), and detection limits (0.4 ng mL^−1^ Se and 0.25 ng mL^−1^ As). TiO_2_@cellulose membranes were applied to water analysis.

**Electronic supplementary material:**

The online version of this article (10.1007/s00604-020-04387-4) contains supplementary material, which is available to authorized users.

**Graphical-abstract:**
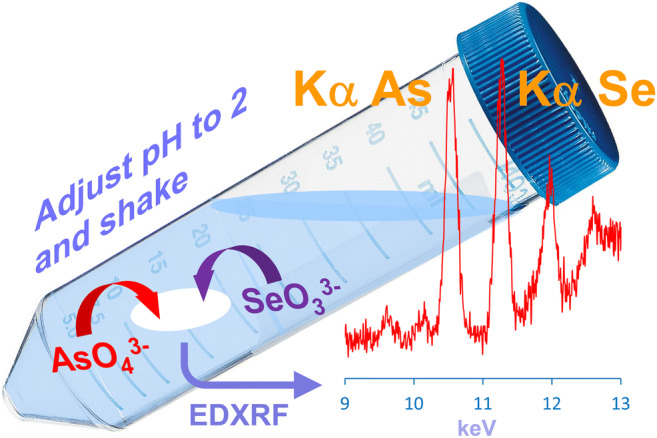
Effective method for determination of ultra trace arsenates and selenites using cellulose-based sorbent

## Introduction

Many sorbents included nanosorbents are mainly oriented towards cationic species of metal but in fact, the sorption of anionic forms is an analytical challenge. Thus, the development of new sorbents dedicated to anionic species of elements is a strategically important and current issue, especially when they are toxic and hazardous for people and natural environment. Arsenic and selenium species can have negative influence on environment. Moreover, arsenic and selenium of various oxidation states have different chemical toxicity. The arsenites and selenates (AsO_3_^3−^, SeO_4_^2−^) are more toxic than the arsenates and selenites (AsO_4_^3−^, SeO_3_^2−^). Therefore, the development of the reliable analytical procedures enabling speciation analysis is very important. References regarding the regulations for water as well as toxicity of arsenic and selenium are given in Electronic Supplementary Material (ESM). Various analytical techniques such as inductively coupled plasma optical emission spectrometry (ICP-OES) [1], graphite furnace atomic absorption spectrometry (GF-AAS) [2, 3] and hydride generation atomic absorption spectrometry (HG-AAS) [4], hydride generation atomic fluorescence spectrometry (HG-AFS) [5, 6], inductively coupled plasma mass spectrometry (ICP-MS) [7, 8], total reflection x-ray fluorescence spectrometry (TXRF) [9], and energy-dispersive x-ray fluorescence spectrometry (EDXRF) [10] were used for the quantification of As and Se at ultra-trace level in different samples. The preconcentration was an important step before determination of these elements. Dispersive liquid-liquid microextraction (DLLME), hollow fiber liquid phase microextraction (HF-LPME), cloud point extraction (CPE), capillary microextraction, and solid phase extraction (SPE) were used for the extraction of As and Se species from various matrices. Numerous adsorbents have been successfully used in both solid-phase extraction (SPE) and micro-SPE (μSPE) for metal ion preconcentration such as modified nanocomposites [11], different types of nanoparticles (carbon, metals, and metal oxides) [12], and cellulose and its derivatives [13]. Extensive studies on new sorbents are still demanded to investigate the sorption of metalloid ions and then determination when different anions are present simultaneously in the solution.

Up to now, not many detailed studies on cellulose modified with metal oxides as sorbents have been described. Certain studies prove that some oxides are characterized by a very high adsorption capacity. The surface of amphoteric titania (TiO_2_) contains hydroxyl groups, and it is a promising material for SPE studies. Determining the nature of active sites susceptible to adsorption of analytes is crucial. In general, the surface hydroxyl groups are prone to the ions adsorption on oxide surfaces [14]. Nevertheless, TiO_2_ is among the few oxides, which after calcined, that has a very low coverage by hydroxyl groups [15]. There are not the systematic data and research relating to the absorption properties as well as using cellulose with titania in the analysis of trace elements. The use of cellulose as a host for TiO_2_ particles is particularly preferred because of its cross-linking character.

In this work, an effective sorbet cellulose-based is developed. The treatment of cellulose fibers is extremely important when using metal oxide particles as a coating, since the properties of the coated substrate varies. TiO_2_ is easy to be synthesized and grafted to cellulose backbone. In this paper, we developed a novel, simple surface treatment method. The cellulose filters were pipetted with Ti(OC_3_H_7_)_4_ and then immersed in ammonia to coat/impregnate cellulose surface with TiO_2_. In this way, using in situ method, new stable filters (TiO_2_@cellulose) of excellent sorptive properties have been obtained. Next, TiO_2_@cellulose was applied as a solid adsorbent in μSPE for preconcentration of metalloid species from an aqueous solution. After sorption, the filters were directly analyzed by an energy-dispersive x-ray fluorescence spectrometry (EDXRF) for determination of preconcentrated trace amount of selenium and arsenic. Direct analysis of filters by EDXRF does not allow the sample to be contaminated and analytes to be lost. The problems mentioned above usually accompany the more complex treatment of the sample. Moreover, as long as analytes are problematic to elute from adsorbents, a great superiority is direct sample analysis. In addition, due to the high selectivity of membranes, they can be used to separate arsenic(V) and selenium(IV) from other ions present in waters.

## Experimental

### Reagents, materials, and apparatus

Propanolan titanium (Ti(OCH_2_CH_2_CH_3_)_4_) 98%, sodium selenite (Na_2_SeO_3_) 99%, and sodium selenate (Na_2_SeO_4_) 98% were purchased from Sigma-Aldrich (Germany); propan-2-ol (C_3_H_7_OH) was purchased from Chempur (Poland); nitric acid (HNO_3_) 65% and ammonia solution (NH_3_·H_2_O) 30% p.a. were purchased from POCH (Poland); standard solutions (1 mg mL^−1^): Se(IV), As(III), and As(V) were purchased from Sigma-Aldrich (Germany), and Cr(III), Cr(VI), Cu(II), Pb(II), Zn(II), and Fe(III) were purchased from Merck (Germany); re-destilated water was from Milli-Q System of Millipore (France); filters Whatman 3MM were purchased from GE Healthcare Life Sciences (United Kingdom); Certified Reference Material (CRM) of natural water, NIST 1640a, was purchased from Sigma-Aldrich (Germany).

The following equipment were used for the research: FEI Nova NanoSEM 230 microscope (FEI Company, Hillsboro, Oregon, USA), x-ray powder diffractometer (XRD) (PANalytical, The Netherlands), energy-dispersive x-ray fluorescence spectrometer (EDXRF) XDV-SD model (Helmut Fischer GmbH, Sindelfingen, Germany), energy-dispersive x-ray fluorescence spectrometer (EDXRF) Epsilon 3 model (PANalytical, Almelo, The Netherlands), and inductively coupled plasma optical emission spectrometer (ICP-OES) SpectroBlue FMS16a model (Spectro Analytical Instruments GmbH, Germany). The apparatus is described in detail in ESM.

### Synthesis of TiO_2_@cellulose membranes

Two hundred microliters of 4% Ti(OC_3_H_7_)_4_ solution in propan-2-ol [16] was pipetted onto a warm cellulose filter (about 60 °C) of diameter 25 mm, in order to cover the whole surface. Then, the filters were dried into the oven at 60 °C (around 30 min), and they were introduced into beakers with NH_3_·H_2_O solution (pH 9–10) and agitated for 1 h. After agitation, the filters were put in an oven for 3 h (60 °C). Then, the filters were cut into smaller ones, thus obtaining 10 mini-filters (5 mm diameter). The mini-filters with this diameter are well matched to spot size of primary x-ray beam emitted by x-ray tube. Consequently, a large part of the adsorbed ions is excited, and therefore, a high analytical signal is obtained.

### Batch adsorption and sample preparation

Experiments aimed at determining adsorption isotherms and developing a preconcentration procedure were preceded by determination of the optimal pH for sorption of analytes. For this purpose, 25 mL single-element aqueous solutions were prepared. Next, obtained mini-TiO_2_@cellulose filters were immersed in these solutions of the desired concentration and pH. The mini-filters were shaken (250 rpm) to reach the equilibrium of adsorption. After this procedure, dried mini-filters at 60 °C were analyzed by EDXRF, and remaining solutions were analyzed by ICP-OES.

## Results and discussion

### Preparation of TiO_2_@cellulose membranes

**Fig. 1:**
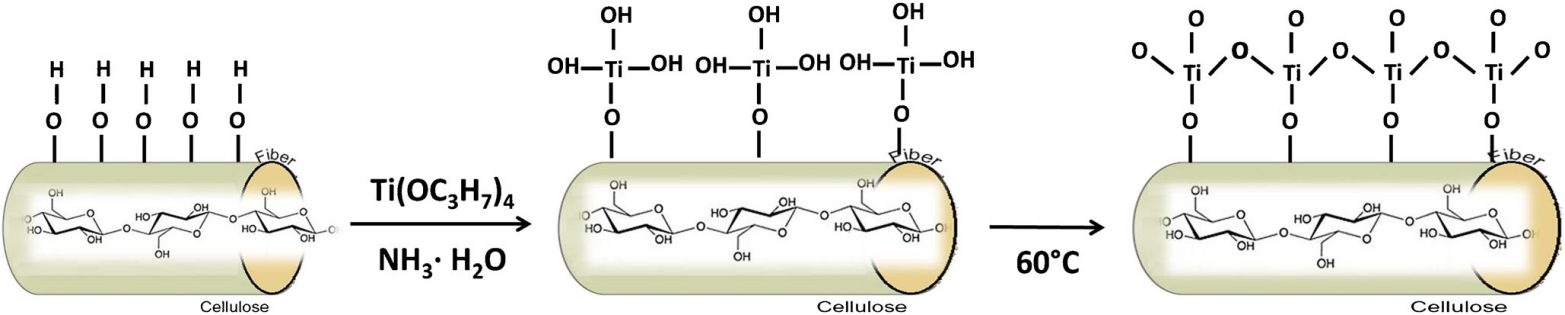
In situ formation of TiO_2_ particles on cellulose filters

The proposed in situ method for developing new cellulose-based sorbents is a simpler method in comparison with the ex situ procedures, in which the metal oxide particles must be synthesized separately and then incorporated in cellulose matrix. Moreover, when using the ex situ methods, the use of retention/covering aids, flocculants, and binders is required. In the in situ methods, metal particles are synthesized on the cellulose matrix directly. The in situ method of sorbent preparation is based on the creation of covalent bonds between TiO_2_ molecules and cellulose. As a result, we obtain a high coverage and homogenous distribution of metal particles on the cellulose surface, as well as high availability of metal particles for sorption. Considering the above facts, as well as the simplicity of filter preparation, the proposed method is more favorable than ex situ methods [17]. The in situ formation of TiO_2_ particles on the cellulose surface (permanent covalent bonds between TiO_2_ and cellulose fibers as result of dehydration reactions between the hydroxyl groups of cellulose and the hydroxyl groups of titania) is demonstrated in Fig. 1**.** The developed method results in a high covering percent of titania particles into the cellulose fibers, which in turn lead to the formation of available active sites for the sorption. Hydrogen bonds can also be formed between hydroxyl groups presented on the surface of cellulose fibers and TiO_2_ particles [18].

The stability of the membranes as well as homogenous distribution of TiO_2_ in membranes is very important in the point of view of effective sorption process as well as XRF analysis. Thus, the influence of the following parameters was examined: temperature of cellulose treatment, application of propan-2-ol, and way of contact ammonia (pipetting, agitating) with membranes covered by Ti(OC_3_H_7_)_4_ solution.

Summarizing this study, the best results we obtained when the preparation of membranes were performed pipetting Ti(OC_3_H_7_)_4_ solution into warm cellulose filter and then agitating the filter with Ti(OC_3_H_7_)_4_ into a beaker with NH_3_·H_2_O solution (pH 9–10). Agitating the filters with the NH_3_·H_2_O solution provides the good contact of entire filter surface with the solution, and, in consequence, homogenous distribution of TiO_2_ on the membranes is observed. After that, the membranes must stay in the oven for 3 h. The longer time (12 h) the filter was left in the oven did not affect the results.

Considering the above facts, filter characteristics as well as the simplicity of filter preparation, the proposed method is interesting and more advantageous than ex situ methods.

### Characterization of TiO_2_@cellulose

**Fig. 2.**
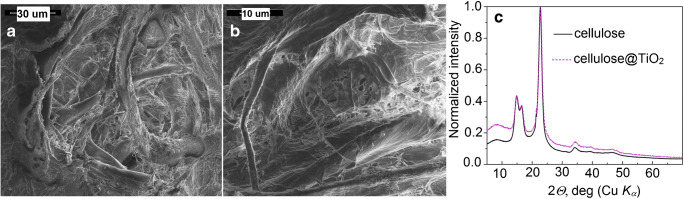
**a**, **b** SEM images of cellulose@TiO_2_. **c** XRD of pure cellulose membrane and modified cellulose @TiO_2_

The TiO_2_@cellulose mini-filters were examined with SEM, XRD, and micro-EDXRF. Figure 2 a, b presents the SEM images of the cellulose@TiO_2_ membrane. The amorphous fraction of TiO_2_ coats cellulose fibers. The spaces between the fibers are also partially filled by the modifier. The results of powder diffraction of pristine and modified cellulose ( Fig. 2c) confirm the amorphous form of TiO_2_. Both diffractograms exhibit the two characteristic broad peaks of cellulose. However, in the modified sample, the diffracted background is significantly higher compared with the pure cellulose, especially for the low diffraction angles. This is an indicator of the additional, amorphous phase present in the diffracted volume of the TiO_2_@cellulose membrane.

Micro-EDXRF mapping is a helpful technique in showing the distribution of the TiO_2_ on the cellulose surface as well as analytes on the TiO_2_@cellulose. The real image of the sorbent surface with actual distribution of analytes was created by analyzing small areas of mini-filters using a suitably small collimator. In Fig. S1, 2D-mapping pictures of TiO_2_@cellulose are presented. As can be seen (see Fig. S1), titanium is distributed over the whole cellulose surface, and the mass per unit area is homogenous. For titanium, the mean mass per unit area is 108.2 ± 8.8 μg cm^−2^.

### Adsorption study

The goal of the pH study is to know which ions are absorbed and which is the best pH for the adsorption of selected oxyanions. According to the results in Fig. S2, both Se(IV) as well as As(V) are adsorbed on TiO_2_@cellulose with the highest recovery (near 100%) at pH = 2. Se(VI) and As(III) are adsorbed with maximum recovery about 10% and 20%, respectively. The Se(VI) is included in the group of oxyanions with moderate or weak affinity, among others, for Al_2_O_3_ and FeOOH [19]. The results of XPS and electrophoretic mobility clearly showed that the bound between selenium(VI) and TiO_2_ is mainly weak electrostatic attraction [20]. Our research also confirms that Se(VI) ions are weakly adsorbed ions on TiO_2_@cellulose in comparison to Se(IV). At pH < 3, Se(IV) is probably adsorbed as H_2_SeO_3_(aq) and HSeO_3_^−^ [21] and As(V) as H_3_AsO_4_ and H_2_AsO_4_^−^ ions [22]. The species for Se(VI), Se(IV), As(V), and As(III) in aqueous solutions at varied pH are presented in Fig. S2.

The surface charge of the adsorbent also depends on the pH. The surface charge is related to what ions (H^+^ or OH^−^) are released during the adsorption process. As(V) adsorption is accompanied by the release of OH^−^ ions, while As(III) adsorption at acidic pH is accompanied by H^+^ release [23]. As long as anion adsorption is associated with the release of OH^−^ ions, better adsorption occurs at a lower pH. The formation of arsenic ion complexes with titanium dioxide is another option. Given the above considerations, As(V) can only form Ti_2_AsO_4_^−^. The interactions between TiO_2_ and H_2_AsO_4_^−^ can be as follows:

$$\equiv \mathrm{Ti}-\mathrm{OH}+{\mathrm{H}}_2{{\mathrm{AsO}}_4}^{-}\to \equiv \mathrm{Ti}-{{\mathrm{H}\mathrm{AsO}}_4}^{-}+{\mathrm{H}}_2\mathrm{O}$$

$$2\equiv \mathrm{Ti}-\mathrm{OH}+{\mathrm{H}}_2{{\mathrm{AsO}}_4}^{-}\to \equiv {\mathrm{Ti}}_2-{{\mathrm{AsO}}_4}^{-}+{2\mathrm{H}}_2\mathrm{O}$$

With bidentate ligands (mono nuclear or binuclear), surface chelates are formed.

The maximum uptake of As(V) occurred at approximately pH 2 (Fig. S2), and it is convergent with the first dissociation constant of H_3_AsO_4_ (pK_a1_ = 2.25). The same situation is observed in case of H_2_SeO_3_ (pK_a1_ = 2.46). This fact confirms statement that pK_a1_ of weak acid adsorbed by metal oxides is close to the pH at which the highest adsorption of this acid is observed [24]. The pK_a1_ of H_3_AsO_3_ is 9.22, and consistently, the growing the adsorption of the As(III) at pH 9 is observed. Thus, the sorption of As(V) and Se(IV) can be explained by similar acidic strength of arsenic acid and selenous acid.

The adsorption of arsenic acid and selenous acid can occur via complexation reactions onto sorbent surface [25]:

$$\equiv \mathrm{Ti}-\mathrm{OH}+{\mathrm{H}}_3{\mathrm{AsO}}_4\to \equiv \mathrm{Ti}-{\mathrm{H}}_{\mathrm{n}}{{\mathrm{AsO}}_4}^{\left(3-\mathrm{n}-1\right)-}+{\mathrm{H}}_2\mathrm{O}+\left(2-n\right){\mathrm{H}}^{+},\mathrm{where}\ n=0,1,2.$$

and analogously

$$\equiv \mathrm{Ti}-\mathrm{OH}+{\mathrm{H}}_2{\mathrm{SeO}}_3\to \equiv \mathrm{Ti}-{\mathrm{H}}_{\mathrm{n}}{{\mathrm{SeO}}_3}^{\left(2-\mathrm{n}-1\right)-}+{\mathrm{H}}_2\mathrm{O}+\left(1-n\right){\mathrm{H}}^{+},\mathrm{where}\ n=0,1.$$

Surface complexation of HSeO_3_^−^ can be illustrated by the following reactions:

$$\equiv \mathrm{Ti}-\mathrm{OH}+{{\mathrm{H}\mathrm{SeO}}_3}^{-}\to \equiv \mathrm{Ti}-{{\mathrm{SeO}}_3}^{-}+{\mathrm{H}}_2\mathrm{O}$$

$$2\equiv \mathrm{Ti}-\mathrm{OH}+{{\mathrm{H}\mathrm{SeO}}_3}^{-}\to \equiv {\mathrm{Ti}}_{2-}{\mathrm{SeO}}_3+{\mathrm{H}}_2\mathrm{O}+{\mathrm{OH}}^{-}$$

The adsorption of Se(IV) and As(V) on TiO_2_@cellulose at pH 2 was simulated using Langmuir and Freundlich isotherm models (for details, see Adsorption isotherms in ESM and Table S1).

The maximum adsorption capacities (q_max_) of our mini-filters determined in this work as well as other single and mixed oxides as adsorbents described in the literature in relation to selenium and arsenic are presented in Table 1.

**Table 1:** Maximum adsorption capacities (q_max_) for selenium(IV) and arsenic(V) by single and mixed oxides

Adsorbent	Species	pH	q_max_, mg g^−1^	Ref.
Fe–Mn hydrous oxides	Se(IV)	4	41.02	[26]
MGO composites	Se(IV)	6–9	23.81	[27]
Mn_3_O_4_ nanomaterial	Se(IV)	4	0.8	[28]
MnFe_2_O_4_ nanomaterial	Se(IV)	4	6.57	[29]
Ce-Mn binary oxide	As(V)	7	63.6	[30]
Zr-doped TiO_2_	As(V)	3	32.4	[31]
Cu doped Fe_3_O_4_	As(V)	5	42.9	[32]
Al_2_O_3_/GO	As(V)	5	43.9	[33]
G/CeO2	Se(IV) As(V)	3 4	14.1 8.4	[34]
GO/CeO_2_	Se(IV) As(V)	3 5	10.7 5.8	[35]
TiO_2_@cellulose	Se(IV) As(V)	2 2	71.1 49.2	This work

As can be seen in Table 1, iron, manganese, cerium, and aluminum oxides, usually as mixed oxides or composites, have been evaluated for selenium and arsenic species adsorption. Maximum adsorption capacities for selenium (IV) are in range of 0.8–64 mg g^−1^. In the light of these results, the obtained maximum sorption capacity of the developed mini-TiO_2_@cellulose is impressively high, i.e., almost twice as high as the highest literature value. In case of arsenic, the maximum sorption capacity of the obtained material is comparable to the others shown in the Table 1, with the exception of composites with cerium oxides, for which the maximum sorption capacities are about 7 times lower than for TiO_2_@cellulose. Comparing the maximum sorption capacity of the obtained membranes with the composite of zirconium ions incorporated into crystal structure of titanium dioxide, we conclude that they are comparable. However, TiO_2_@cellulose also gives the possibility of adsorption of Se (IV) at the same pH. Thus, its use is wider, because at the same time, we can absorb and then determine both As(V) as well as Se(IV). In this way, we save time, the amount of sorbent and analyzed sample, and we do not generate additional wastes. This aspect of the developed method is therefore extremely important from the point of view of environmental protection, as well as compliance with the principles of green analytical chemistry.

The contact time of membrane with analyte solution can significantly affect the adsorption of analytes percentage, particularly when high sample volume is used. The influence of contact time and the sample volume on the adsorption of Se(IV) and As(V) were studied in the range of time, 5–180 min and sample volume, 20–100 mL. Figure S3 shows that quantitative adsorption of both Se(IV) as well as As(V) is achieved then the shaking time was at least 120 min. It should be also emphasized that even when the membranes were vigorously shaken (250 rpm) in an aqueous solution for 180 min, no damage was noted. This indicates a high durability and stability of the obtained membranes. When considering sorption time of developed method, it can get the impression that it is quite long and may be considered a slight drawback. However, since the method involves only shaking the filters in the analyte solution, nothing stands in the way to prepare many samples at one time using an orbital platform shaker. Simultaneous sample preparation is therefore very beneficial, especially if the method is not labor intensive.

### Interferent study

Considering the coexisting cations, anions and natural organic matter (NOM) included humic acid (HA) in real water samples, and the influence of Na^+^, K^+^, Mg^2+^, Ca^2+^, Al^3+^, Fe^3+^, SO_4_^2−^, PO_4_^3^, Cl^−^, NO_3_^−^, and HA on the recovery of Se(IV) and As(V) adsorbed on TiO_2_@cellulose was investigated. The obtained results are shown in Table S2 and Fig. S4. The high tolerance of TiO_2_@cellulose for foreign ions is observed. Therefore, it can be concluded that the obtained mini-membranes can be used to quantify trace amounts of arsenic and selenium, in presence of many another ions in water, by EDXRF in a simple and straightforward manner.

### Validation parameters

**Fig. 3:**
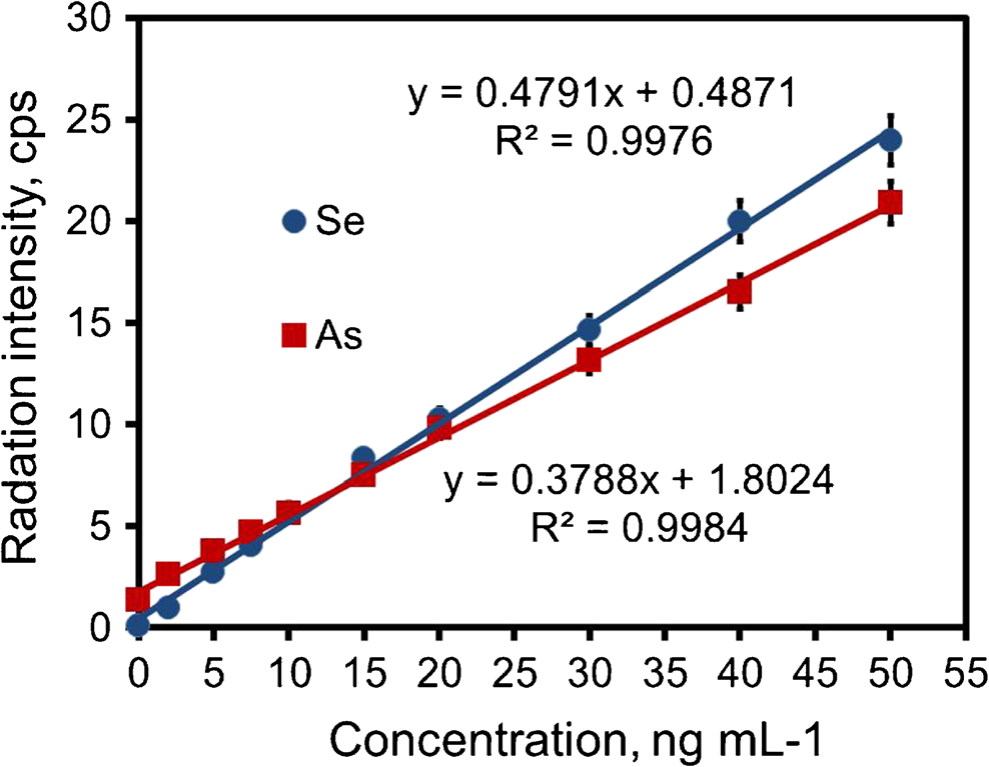
Calibration curves of the signal intensities vs the concentrations of Se and As

The analytical figures of merit of developed μSPE/EDXRF are presented in Table 2 and Fig. 3. The calibration plot is reflected in a correlation coefficient *R* of 0.999 and 0.999 for Se(IV) and As(V), respectively. The limit of detection (LOD) and limit of quantification (LOQ) were calculated from the following equations: LOD = (3·k^−1^)·(R_B_·t^−1^)^1/2^, where k is the sensitivity of the method, *R*_B_ is the background count rate in counts·s^−1^, and *t* is the counting time, and LOQ = 3·LOD. The good LODs mean 0.4 ng mL^−1^ and 0.25 ng mL^−1^ for selenium and arsenic, respectively, characterized developed method.

**Table 2:** Analytical figures of merit of the μSPE/EDXRF using TiO_2_@cellulose as solid adsorbent. Measurement conditions: Rh x-ray tube operated at 30 kV and 300 μA, 300 s counting time, air atmosphere, and Ag primary beam filter

Parameter	Se(IV)	As(V)
Linearity, ng mL^−1^	2–50	2–50
Correlation coefficient *R*	0.999	0.999
Sensivity, mL ng^−1^ s^−1^	0.479	0.379
LOD, ng mL^−1^	0.4	0.25
LOQ, ng mL^−1^	1.2	0.75
RSD, %	4.5	4.5

Please note that the LODs are 125 and 40 times lower for selenium and arsenic, respectively, than the acceptable maximum contaminant levels (MCL) of these elements. MCL of 50 ng mL^−1^ (for Se) and 10 ng mL^−1^ (for As) is established by US EPA.

**Fig. 4:**
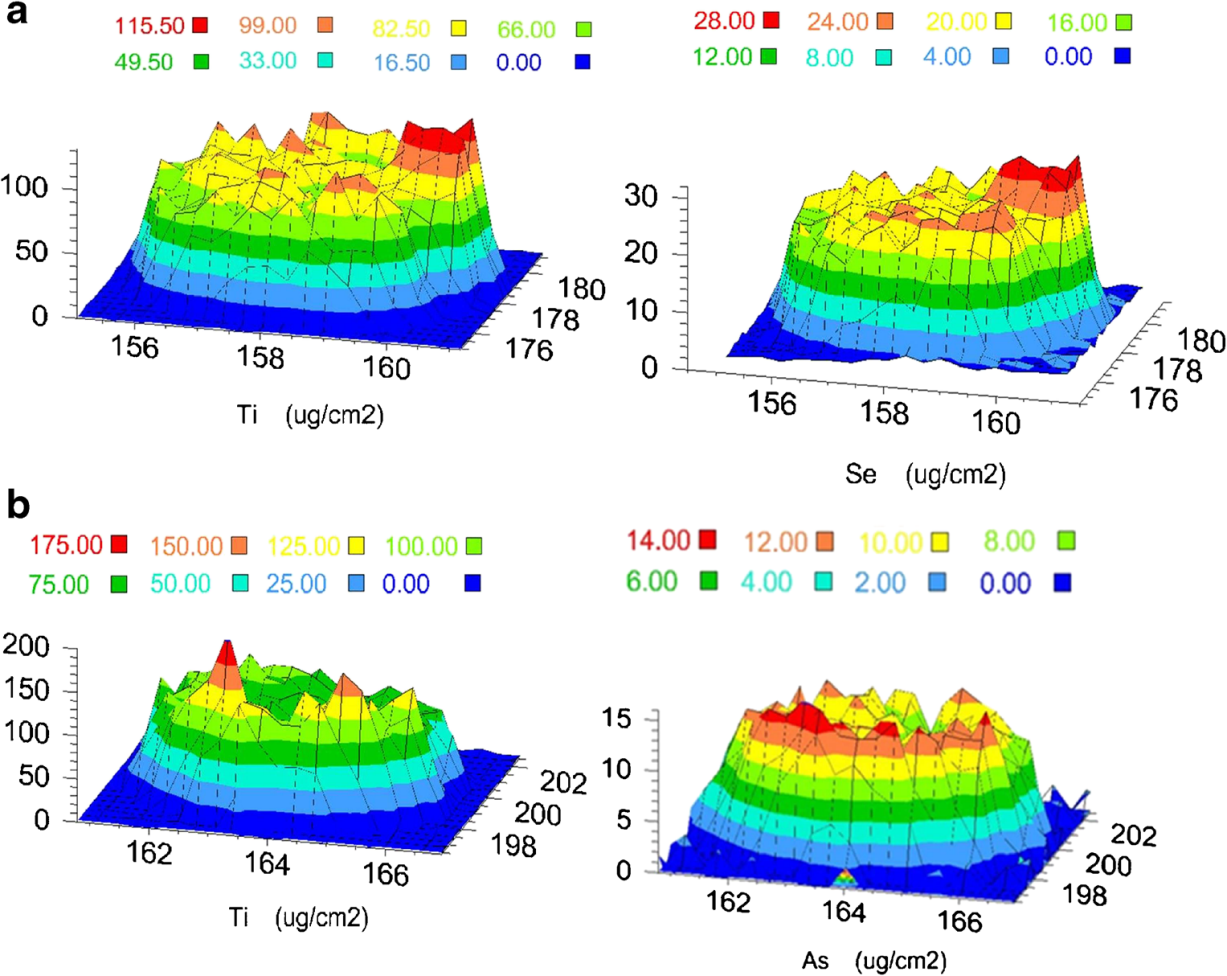
Mapping of TiO_2_@cellulose with adsorbed Se(IV) or As(V). **a** Distribution patterns of Ti and Se(IV) on TiO_2_@cellulose. **b** Distribution patterns of Ti and As(V) on TiO_2_@cellulose

The method precision expressed by the relative standard deviation (RSD) is 4.5% for both analytes. In case of trace analysis, this value should be considered as very good. Good precision results *inter alia* from the high homogeneity of the surface of the analyzed samples. As illustrated in Fig. 4, the mapping of TiO_2_@cellulose with analytes confirms that the distribution of selenium as well as arsenic is homogenous in the whole TiO_2_@cellulose surface. The mappings in Fig. 4 clearly show that the distribution of titanium and selenium or titanium and arsenic is overlapping. The distribution of elements expressed as the mass per unit area for Ti is higher than for Se and As. This fact resulted from the differences in concentration of Ti and Se or As in the analyzed samples. Titanium as TiO_2_ beside cellulose is the main component of analyzed membranes. The high titanium concentration allows covering whole cellulose surface. Although the concentration of selenium and arsenic is at trace level, the distribution of selenium as well as arsenic is homogeneous. This fact shows that the metalloid sorption occurs evenly over the entire membrane surface.

In Tables 3 and 4, the methods of determination of Se(IV) and As(V) using various adsorbents based on cellulose membranes are compared, respectively.

**Table 3:** The determination of selenium species with various adsorbents based on cellulose membranes

Analyte	pH	Sorbent	Mass of sorbent, mg	Eluent	LOD, ng∙mL^−1^	RSD, %	Technique detection	Ref.
Se(IV)	3	Graphene membrane /APDC^a^	0.45	–	0.15	2.3	TXRF^c^	[9]
Se(IV)	4.5	TMA-C^b^	20	3.0 mol/L HCl-2% KClO_3_	–	2	ICP-OES^d^	[1]
Se(IV)	6	La(OH)_3_ coated on cellulose fiber	5	1.0% NaBH_4_ (in 1.0% NaOH)	0.009	1.7	HG-AFS^e^	[5]
Se(IV) Se(VI)		Cellulose TLC plate	–	–	3 2	3 3	DLTV-ICP-MS^f^	[8]
Se(IV)	2.0	TiO_*2*_@ cellulose	0.25*	Solvent-free	0.4	4.5	EDXRF	This work

*mass of TiO_2_

^a^Ammonium pyrrolidinedithiocarbamate

^b^Cellulose grafted thiomalic acid

^c^Total reflection x-ray fluorescence spectrometry

^d^Inductively coupled plasma-optical emission spectrometry

^e^Hydride generation atomic fluorescence spectrometry

^f^Diode laser thermal vaporization inductively coupled plasma mass spectrometry

**Table 4:** The determination of arsenic species with various adsorbents based on cellulose membranes

Analyte	pH	Sorbent	Mass of sorbent, mg	Eluent	LOD, ng∙mL^−1^	RSD, %	Technique detection	Ref
As(III)	3	Graphene membrane/APDC^a^	0.45	–	0.2	4.1	TXRF^d^	[9]
As(V)	6	MESM^b^	10	1.5 mol L^−1^ HCl	0.015	3.5	HG-AFS^e^	[6]
As(III)	1	cellulose/SiO_2_/MPTMS^c^	4.4	–	0.045	5.6	EDXRF^f^	[10]
As(V)	2.0	TiO_**2**_@ cellulose	0.25*	Solvent-free	0.25	4.5	EDXRF	This work

*mass of TiO_2_

^a^Ammonium pyrrolidinedithiocarbamate

^b^Methyl esterified egg-shell membrane

^c^Silica coating cellulose fibers modified with (3-mercaptopropyl)-trimethoxysilane

^d^Total reflection x-ray fluorescence spectrometry

^e^Hydride generation atomic fluorescence spectrometry

^f^Energy-dispersive x-ray fluorescence spectrometry

Three basic advantages of the developed method should be emphasized, looking at the others presented in the Tables 3 and 4, i.e., (1) a very low mass of the sorbent used, which is the smallest one among masses used in all other methods; (2) no need to elute the analytes after sorption and before the determination; and also (3) the possibility of simultaneous determination of both selenium and arsenic in one sample. In case of method using graphene membrane and cellulose/SiO_2_/MPTMS, the analytes did not have to be also eluted, but the mass of sorbents was about 2 and 15 times bigger, and additionally, in case of the first mentioned sorbent, it was necessary to use a chelating agent (APDC) for sorption of analytes. The method with cellulose TLC plate and DLTV-ICP-MS detection was performed without elution of analytes, but LODs obtained then were about 10 times worse in comparison with developed method. As can been seen in Tables 3 and 4, the value of LOD depends on detection technique. The lowest LODs were achieved using HG-AFS. However, in case of EDXRF with very low-power x-ray tube (9 W) without gas consumption, LODs obtained for analytes should be considered as very good. Therefore, taking all this into account, the developed method coupled with EDXRF analysis can be considered as new, promising, and competitive compared with previous ones and also environmentally friendly, lying in the green principles of analytical chemistry.

### Application

The trustworthiness of the developed procedure was established on the basis of the analysis of various types of drinking waters. The studies were performed using waters spiked with analytes at two concentration levels (7.5 and 10 ng·mL^−1^). As demonstrated in Table S3, for all the studied water matrices, recoveries near 100% were assessed. Since TiO_2_@cellulose is highly selective towards Se(IV) and As(V) at pH 2, the speciation analysis can also be performed (see Table S4). The total concentration of Se was calculated after reduction of Se(VI) to Se(IV) with H_2_SO_4_ and C_2_H_5_OH. The total concentration of As was calculated after oxidation of As(III) to As(V) with KMnO_4_. Then, the Se(VI) and As(III) content was calculated as the difference between total Se and Se(III) or As(V) concentration, respectively. The results presented in Table S4 indicate that method can be successfully applied for speciation of selenium and arsenic species in real water samples with good recovery and precision.

The developed method was used to determine selenium and arsenic in the CRM of natural water (NIST 1640a) to assess its accuracy. The results of this analysis are shown in Table 5. The high compatibility of certified values with determined analyte concentrations supports the suitability of the TiO_2_@cellulose membrane in routine ultratrace analysis. The error of determination of analytes with developed method is about 5.5%, and recovery is near 100%.

**Table 5:** Analysis of CRM (NIST 1640a) (± std.; *n* = 3)

Analyte	Certified concentration, μg L^−1^	Obtained concentration, μg L^−1^	Error, %	Recovery, %	*t* test (*t*_0.05_ = 2.92)
As	8.075 ± 0.070	8.5 ± 0.52	5.2	105	1.42
Se	20.13 ± 0.17	18.9 ± 0.83	6.1	94	2.57

Concentration of minor elements in mg L^−1^: Ca (5.615 ± 0.021), Mg (1.0586 ± 0.0041), K (0.5799 ± 0.0023), Si (5.210 ± 0.021), and Na (3.137 ± 0.031); concentration of trace element in μg L^−1^s: Al (53.0 ± 1.8), Ba (151.80 ± 0.83), B (303.1 ± 3.1), Cr (40.54 ± 0.30), Co (20.24 ± 0.24), Cu (85.75 ± 0.51), Fe (36.8 ± 1.8), Mn (40.39 ± 0.36), Mo (45.60 ± 0.61), Ni (25.32 ± 0.14), Pb (12.101 ± 0.050), Sr (126.03 ± 0.27), U (25.35 ± 0.27), V (15.05 ± 0.25), and Zn (55.64 ± 0.35)

## Conclusion

This research was focused on the development eco-friendly cellulose-based membrane (TiO_2_@cellulose). The incorporation of TiO_2_ particles into raw cellulose filter by developed in situ method is more beneficial than ex situ procedures. TiO_2_@cellulose membranes obtained in this way are characterized by high maximum adsorption capacity, excellent stability in aqueous solutions, and high homogeneity. The selectivity of TiO_2_@cellulose membranes towards Se(IV) and As(V) indicates their potential application in speciation of these metalloids. Taking advantage of the excellent properties of membranes, a simple method for determining trace amounts of selenium and arsenic in water, was developed. The simultaneous adsorption/determination of Se(IV) and As(V) at the same pH using very low mass of adsorbent and without the need for using additional reagents, such as a chelating agent, is profitable. Even though the adsorption time can be considered as not very short, still as long as the orbital platform shaker is applied, the procedure is not labor-intensive and demanding since many samples are prepared at once. Because the ultratrace amounts of metalloid species are simultaneously and directly determined onto the mini-membranes without the need for eluting analytes, the quantitative analysis is not only easy and cost-effective, but also fulfilling the green analytical chemistry principles.

## Electronic supporting material (ESM)

ESM 1 (PDF 705 kb)
