# Supplementary material for: Cellulose mini-membranes modified with TiO2 for separation, determination, and speciation of arsenates and selenites
Source: Mikrochim Acta. 2020 Jul 6;187(8):430. doi: 10.1007/s00604-020-04387-4 (PMC7338822; doi:10.1007/s00604-020-04387-4)
Supplement: ESM 1 — (PDF 705 kb) [file 604_2020_4387_MOESM1_ESM.pdf]

Electronic Supporting Material
on the Microchimica Acta publication entitled

Cellulose mini-membranes modified with TiO_2 for separation, determination and speciation of arsenates and selenites

Beata Zawisza[*a], Rafal Sitko[a], Ignasi Queralt[b], Eva Margui[c], Anna Gagor[d]

[a] University of Silesia, Institute of Chemistry, Szkolna 9, 40-006 Katowice, Poland
[b] Institute of Environmental Assessment and Water Research, Dep. of Geosciences, IDAEA-CSIC, Jordi Girona St., 18-26, 08034 Barcelona, Spain
[c] Department of Chemistry, University of Girona, Faculty of Sciences, C/M.Aurèlia Campmany 69, Girona, Spain
[d] Institute of Low Temperature and Structure Research, Polish Academy of Sciences, P.O. Box 1410, 50-950 Wrocław, Poland

* Corresponding author. E-mail address: beata.zawisza@us.edu.pl ; orcid.org/0000-0001-6582-2839

Apparatus

SEM micrographs were analysed using FEI Nova NanoSEM 230 microscope. X-ray powder diffraction data (XRD) (PANalytical, The Netherlands) were collected on X'Pert PRO X-ray diffractometer with PIXcel ultrafast line detector and Soller slits for Cu K_{α} radiation. The measurements were performed in Bragg-Brentano geometry.

Powder diffraction data (XRD) were collected on X'Pert PRO X-ray diffractometer with PIXcel ultrafast line detector and Soller slits for Cu K_{α} radiation. The measurements were done in Bragg-Brentano geometry.

A commercial EDXRF system (XDV-SD model, Helmut Fischer GmbH, Sindelfingen, Germany) equipped with a small spot X-ray beam (aperture collimators of 0.1, 0.3, 1.0 and 3.0 mm) was used to study the spatial distribution (homogeneity) of the membranes. The main features of this equipment are a low-power tungsten X-ray tube operating at a maximum power of 50W (fixed voltages of 10, 30 and 50 kV and current values of 0.1–1 mA) and a SD semiconductor detector (Peltier cooled to −50 °C) with a resolution of 180 eV at Mn Kα. This spectrometer is also equipped with four primary filters (Ni, 10 µm; Al, 500 and 1000 µm; Ti, 300 µm and Mo, 70 µm). In this configuration, a hole mirror located in the path of the primary beam allows the measurement spot to be precisely selected using a colored video camera and the area to be viewed on a PC monitor during the measurement procedure. Fully automatic measurements can be conducted using the XYZ-programmable motorized stage. The instrument is controlled by WinFTM®-v.6.35 software, which is used for both the acquisition of spectra and the treatment of the spectral data. The software was initially designed for the simultaneous analysis of layer thickness and composition with application to single layers or multi-layered materials. The fundamentals of this software are detailed in the Roessiger and Nensel work [1]. This software program utilizes for the evaluation of the measured spectrum the Fundamental Parameters (FP) approach [2], with the support of pure elements spectral data, in a first approach.

Energy-dispersive X-ray fluorescence spectrometry (EDXRF) measurements were performed using Epsilon 3 (PANalytical, Almelo, The Netherlands) with a Rh target X-ray tube with a 50 μm Be window and max. power of 9 W. The spectrometer is equipped with a thermoelectrically cooled silicon drift detector (SDD) with 8 μm Be window and resolution of 135 eV at 5.9 keV. The spectrometer is equipped with spinner and five primary filters that can be selected to improve measuring conditions for determined elements.

Inductively coupled plasma optical emission spectroscopy (ICP-OES) (Spectro Analytical Instruments GmbH, Germany) measurements were performed using a SpectroBlue FMS16a spectrometer with inductively coupled plasma (ICP) excitation (Spectro Analytical Instruments) and a charge coupled device detector. The following

operation parameters were used for measurements: plasma power – 1.45 kW; coolant gas – Ar, 12 L min^{-1}; auxiliary gas – Ar, 1 L min^{-1}; nebulizer gas – Ar, 1 L min^{-1}; nebulizer pressure – 3.2 bar; nebulizer-cross-flow type; sample uptake rate – 2 mL min^{-1}; wavelength – 193.759 nm, 196.090 nm, 324.754 nm and 220.353 nm for As, Se, Cu and Pb, respectively.

The regulations for water as well as toxicity of arsenic and selenium are given by United States Environmental Protection Agency (USEPA) [3,4], World Health Organization (WHO) [5], European Union (EU) [6]. Toxicity of arsenic and selenium are described *inter alia* by [7,8]. Maximum contaminant levels (MCL) of arsenic was established by USEPA at 10 ng mL^{-1}. USEPA established also the maximum acceptable concentration level of selenium in surface waters at 5.0 µg/L. As reported by WHO, EU, and USEPA selenium limit in drinking water was set at 40 µg L^{-1}, 10 µg L^{-1} and 50 µg L^{-1}, respectively.

Characterization of TiO_2@cellulose

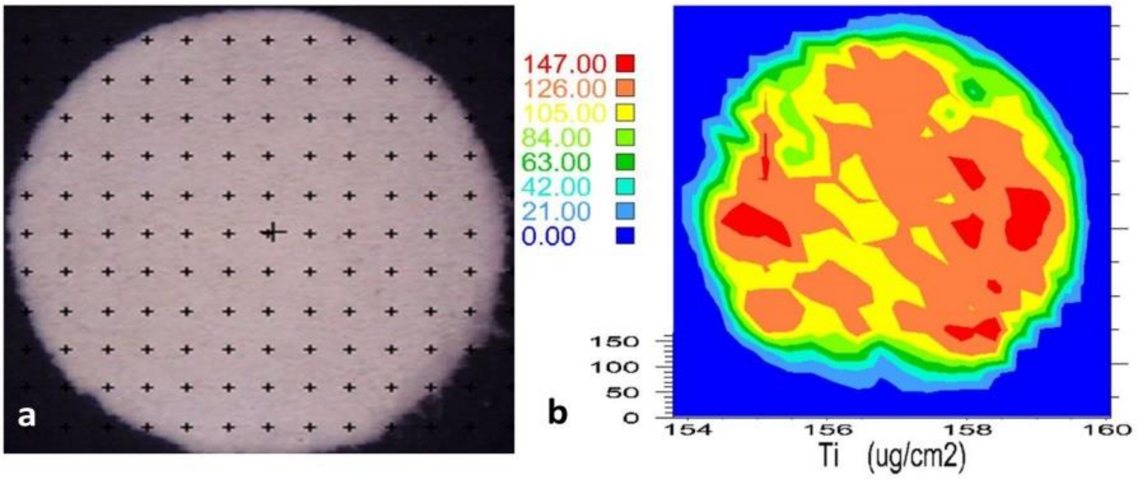

Figure S1. Mapping of TiO_2@cellulose (collimator - 0.3 millimeter diameter).

Adsorption study

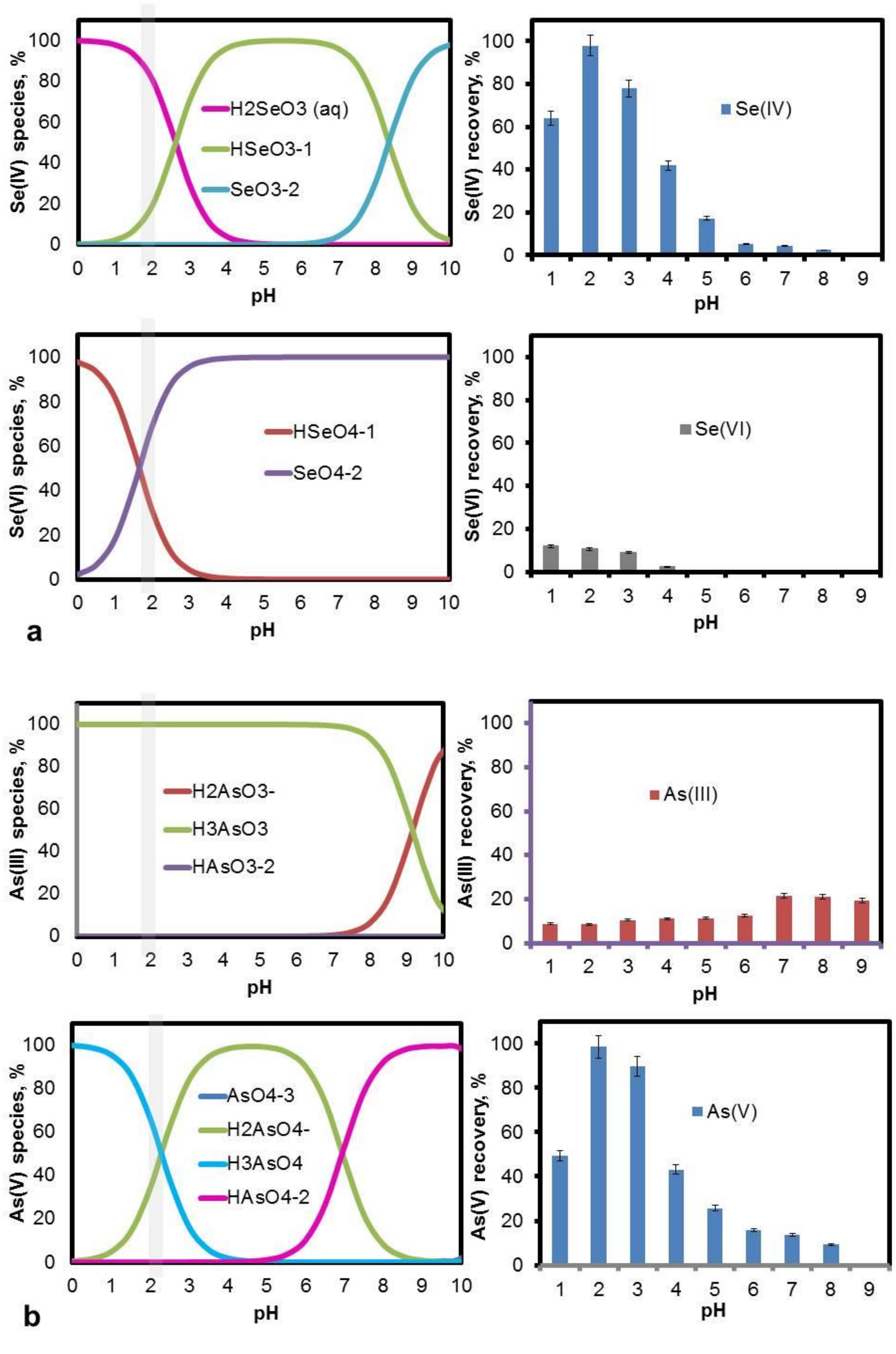

Figure S2. The dependence of Se and As species on pH and the effect of pH on the recovery of Se(IV), Se(VI) and As(III), As(V) on TiO_2@cellulose.

Adsorption isotherms

The adsorption of Se(IV) and As(V) ions on TiO_2@cellulose at pH 2 was simulated using Langmuir [9,10] and Freundlich [11] isotherm models:

$$q_e = \frac{q_{\max} K_L C_e}{1 + K_L C_e}$$

$$q_e = K_F C_e^{1/n}$$

where q_{max} is the maximum amount of ions adsorbed per unit weight of TiO_2 at the high equilibrium ion concentration (mg g^{-1}), K_L is the constant related to the free energy of adsorption (L mg^{-1}), C_e is the equilibrium concentration, which is achieved during stabilization of the system (mg·L^{-1}), K_F (mg^{1-n} L^n g^{-1}) and n are Freundlich constants related to the adsorption capacity and the adsorption intensity, respectively.

Table S1. The list of parameters for Langmuir and Freundlich models.

	Langmuir			Freundlich		
	q_{max}	K_L	R	K_F	n	R
Se(IV)	71.1	3.49	0.996	46.8	3.04	0.987
As(V)	49.2	2.58	0.999	27.7	1.41	0.981

Isotherm parameters are included in **Table S1**. The better fitting the adsorption equilibrium data to the Langmuir model than the Freundlich model suggests a chemical adsorption process.

The influence of contact time and the sample volume on the adsorption of Se(IV) and As(V).

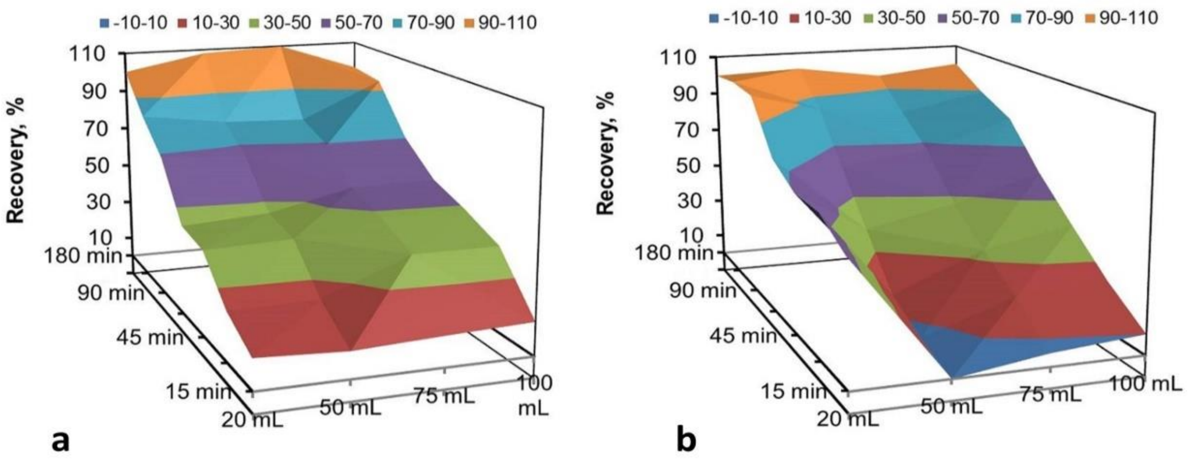

Figure S3. Effect of sample volume and contact time for TiO_2@cellulose and Se(IV) (a) as well as As(V) (b) (x and z axis are sample volume and contact time, respectively).

Interferent study

The concentrations of potential interferents were chosen according to a literature study [3]: 20-200 μg mL^{-1} (Na^+, K^+, Mg^{2+}, Ca^{2+}), 0.5-10.0 μg mL^{-1} (Al^{3+}, Fe^{3+}), 0.01-250 μg mL^{-1} of SO_4^{2-}, 0.01-1.0 μg mL^{-1} of PO_4^{3-}, 100-500 µg mL-1 of Cl^-, 100-800 μg mL^{-1} of NO_3^- and 0.5 to 5.0 μg mL^{-1} of HA. The obtained results (see **Table S2** and **Figure 4S**) prove that the metal cations, such as: Na^+, K^+, Mg^{2+}, Ca^{2+}, Al^{3+} as well as anions such as: Cl^-, NO_3^-, SO_4^{2-} and HA in the whole studied concentration range does not influence the analytes sorption, which are adsorbed with recovery values in the range of 92-108 %. Only, the impact of Al^{3+} and SO_4^{2-} at high concentration levels (10 μg mL^{-1} Al^{3+} and 250 μg mL^{-1} SO_4^{2-}) on arsenic sorption can be observed (Recovery of

85 %, and 62 %, respectively). Reducing the Al^{3+} and SO_4^{2-} concentration to 5 μg mL^{-1} and 50 μg mL^{-1}, respectively, allows obtaining 99-95% recovery of As(V). However, the US EPA recommends that aluminum levels for secondary drinking water do not exceed of 200 µg L^{-1} so, it is not a critical issue for As(V) determination in this type of water. Sulfate is not classified under the primary standards for drinking water. The secondary maximum contaminant level (SMCL) for sulfate is 250 μg mL^{-1}. The influence of 2.5 μg mL^{-1} Fe^{3+} the recovery of both analytes is not observed. SMCL for iron is 0.3 μg mL^{-1}. The concentration of phosphates in the water sample should not exceed the concentration of 0.25 μg mL^{-1} (secondary standards) and 0.1 μg mL^{-1} (primary standards), so that the impact on recovery of As and Se is not observed. However, the maximum acceptable level of phosphorous in water to avoid accelerated eutrophication is 0.1 μg mL^{-1}. Sum up, the acidic solution (pH 2) enables the quantitative adsorption of Se (IV) and As (V), while preventing the precipitation of some elements found in real waters.

Table S2. Effect of potential interferences on recovery of Se(IV) and As(V) on TiO_2@cellulose.

Interferent	Concentration, µg mL^{-1}	Added as	Interferent/ analyte ratio	Recovery± RSD, %	
				As(V)	Se(IV)
Na^+	200	$NaNO_3$	20 000	100.5±0.38	107.8±0.38
K^+	200	KNO_3	20 000	98.64±0.07	96.7±0.10
Mg^{2+}	200	$Mg(NO_3)_2 \cdot 6H_2O$	20 000	91.91±0.09	95.5±0.36
Ca^{2+}	200	$Ca(NO_3)_2 \cdot 4H_2O$	20 000	105.4±0.50	99.3±0.55
Al^{3+}	10	$Al(NO_3)_3 \cdot 9H_2O$	10 000	85.2±0.34	99.4±0.23
Al^{3+}	5	$Al(NO_3)_3 \cdot 9H_2O$	5 000	99.3±0.34	99.31±0.25
Fe^{3+}	2.5	$Fe(NO_3)_3 \cdot 9H_2O$	250	100.05±0.07	104.8±0.58
NO_3^-	800	KNO_3	80 000	97.8±0.59	97.0±0.41
SO_4^{2-}	250	Na_2SO_4	25 000	61±1.17	104.8±0.72
SO_4^{2-}	50	Na_2SO_4	5 000	95.0±0.77	103.9±0.85
PO_4^{3-}	0.25	$Na_3PO_4 \cdot 12H_2O$	25	84.2±0.87	97.4±0.94
PO_4^{3-}	0.1	$Na_3PO_4 \cdot 12H_2O$	10	93.3±0.97	97.8±0.51
Cl^-	500	NaCl	50 000	102.4±0.49	95.8±0.29
HA	5.0	Humic acid	500	97.4±0.11	100.7±0.53

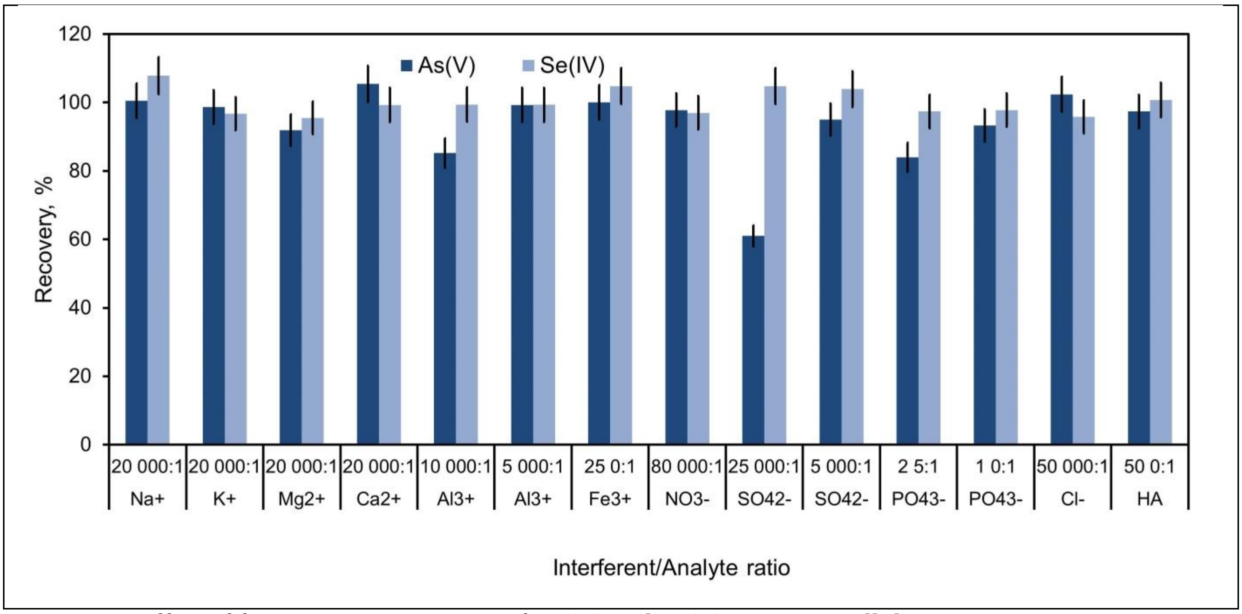

Figure S4. Effect of foreign ions on recovery of Se(IV) and As(V) on TiO_2@cellulose.

Application

Table S5. Analyte determination in waters spiked with Se(IV) or As(V) (± std; *n* = 3)

Sample	Added, ng·mL^{-1}	Found, ng·mL^{-1}		Recovery, %	
	Se(IV) or As(V)	Se(IV)	As(V)	Se(IV)	As(V)
Tap water	0	< LOD	< LOD		
	7.5	7.3 ± 0.16	6.9 ±0.18	97.3	92.0
	10	9.7 ± 0.11	9.1 ±0.21	97.0	91.0
Spring water	0	< LOD	< LOD		
	7.5	7.0 ± 0.11	6.9 ±0.23	93.3	92.0
	10	9.4 ± 0.15	9.2 ±0.24	94.0	92.0
Mineral water	0	< LOD	< LOD		
	7.5	7.6 ± 0.29	7.2 ±0.32	101.3	96.0
	10	9.7 ± 0.19	9.4 ±0.31	97.0	94.0

Table S6. Determination of Se as well as As species in water (± std; n = 3)

Added, ng mL^{-1}				Found, ng mL^{-1}				Recovery, %			
Se(IV)	Se(VI)	As(III)	As(V)	Se(IV)	Se(VI)*	As(III)**	As(V)	Se(IV)	Se(VI)	As(III)	As(V)
0	0	0	0	< DL	< DL	< DL	< DL	-	-	-	-
10	0	10	0	10.4±0.71	< DL	9.7±0.33	< DL	104	-	97	-
0	10	0	10	< DL	9.0±0.63	<DL	10.3±0.60	-	90	-	103
10	10	10	10	9.2±0.58	8.9±0.67	9.4±0.29	9.7±0.40	92	89	94	97

*Se(VI) = Se(total)-Se(IV); **As(III) = As(total)-As(V)

References

1. Roessiger V, Nensel B (2006) Analysis of layers. In: Beckhoff B, Kanngiesser B, Langhoff, N, Wedell R, Wolff H (Eds.), Handbook of practical X-ray fluorescence analysis. Springer-Verlag, Berlin Heidelberg, pp. 554-600
2. Thomsen V (2007) Basic Fundamental Parameters in X-Ray Fluorescence. Spectroscopy 46: 46-50
3. USEPA (2009) National Primary Drinking Water Regulations, List of Contaminants and their (MCLs). EPA 816-F-09-0004. United states Environmental Protection Agency, Washington DC
4. USEPA (2014) External Peer Review Draft — Aquatic Life Ambient Water Quality Criterion For Selenium — Freshwater, EPA-820-F-14-005. United states Environmental Protection Agency. Washington, DC
5. WHO (2011) Guidelines for Drinking-water Quality. 4th ed. Geneva
6. European Commission (1998) COUNCIL DIRECTIVE 98/83/EC on the quality of water intended for human consumption. In: 330 L (Ed.) Official Journal of the European Communities. pp. 32–54.
7. Hughes M F (2002) Arsenic toxicity and potential mechanisms of action. Toxicol Lett 133: 1-16
8. Wake B D, Bowie A R, Butler E C V, Haddad P R (2004) Modern preconcentration methods for the determination of selenium species in environmental water samples. TrAC Trends Anal Chem 23: 491–500
9. Langmuir I (1916) The Constitution and fundamental properties of solids and liquids. Part I. Solids. J Am Chem Soc 38: 2221–2295
10. Langmuir I (1918) The adsorption of gases on plane surfaces of glass, mica and platinium. J Am Chem Soc 40: 1361–1403
11. Freundlich, H M F (1916) Uber die Adsorption in Lasugen. Z Phys Chem 57: 385–470
